# Antifungal susceptibilities of opportunistic filamentous fungal pathogens from the Asia and Western Pacific Region: data from the SENTRY Antifungal Surveillance Program (2011–2019)

**DOI:** 10.1038/s41429-021-00431-4

**Published:** 2021-06-30

**Authors:** Michael A. Pfaller, Cecilia G. Carvalhaes, Paul Rhomberg, Shawn A. Messer, Mariana Castanheira

**Affiliations:** 1 JMI Laboratories, North Liberty, IA USA; 2 University of Iowa, Iowa City, IA USA

**Keywords:** Drug development, Medical research

## Abstract

Antifungal surveillance is an important tool to monitor the prevalence of uncommon fungal species and increasing antifungal resistance throughout the world, but data comparing results across several different Asian countries are scarce. In this study, 372 invasive molds collected in the Asia-Western Pacific region in 2011–2019 were susceptibility tested for mold-active triazoles (isavuconazole, posaconazole, voriconazole, and itraconazole). The collection includes 318 *Aspergillus* spp. isolates and 53 non-*Aspergillus* molds. The MIC values using CLSI methods for isavuconazole versus *Aspergillus fumigatus* ranged from 0.25 to 2 mg l^−1^. Isavuconazole, itraconazole, posaconazole, and voriconazole acted similarly against *A. fumigatus*. The mold-active triazoles exhibited a wildtype phenotype to most of the *Aspergillus* spp. isolates tested (>94%), but poor activity against *Fusarium solani* species complex and *Lomentospora prolificans*. Voriconazole was most active against the *Scedosporium* spp. and posaconazole was most active against the Mucorales. In summary, isavuconazole displayed excellent activity against most species of *Aspergillus* and was comparable to other mold-active triazoles against non-*Aspergillus* molds.

## Introduction

Invasive mold infections (IMI) threaten to limit the life-saving advances of modern medical technology [1–3]. The genera *Aspergillus*, *Fusarium*, *Lomentospora*, *Scedosporium*, and molds belonging to the order Mucorales have been termed the “Big Five” mold killers of humans [3]. Whereas much of the literature concerning the species distribution and antifungal resistance profiles of the species causing IMI has focused on isolates from North America and Europe [4–7], the greatest burden of fungal disease in the world resides in the Asia and the Western Pacific (APAC) region [2, 8]. Numerous factors specifically contribute to the excess fungal diseases in APAC, including a tropical environment in much of the region, inadequately trained healthcare personnel, overuse of steroids and antimicrobials, healthcare practices that are compromised by underfunding, and excessive patient loads in public sector hospitals [8]. Further compromising the diagnosis and treatment of IMI in APAC is the lack of high-quality microbiology laboratories and a limited awareness of fungal disease [8]. Although conventional microscopy and culture are available in most settings, few laboratories perform nucleic acid sequencing or matrix-assisted laser desorption ionization-time of flight mass spectrometry (MALDI-TOF MS) for fungal identification and antifungal susceptibility testing is rarely performed on filamentous fungi [8]. These limitations highlight the need for quality laboratory support, training in medical mycology, and improved access to modern medical technology to facilitate the diagnosis and treatment of IMI in the APAC region [8].

Antifungal resistance is increasingly noted among filamentous fungi throughout the world [2, 4, 6, 9–12]. Although acquired resistance to the triazole antifungal agents in isolates of *Aspergillus fumigatus* has been seen largely in European isolates [5, 7, 13], both acquired and intrinsic resistance among *Aspergillus* and other less common molds have been reported from the APAC region [2, 8, 9, 12, 14, 15]. Given the unpredictable susceptibility of emerging molds to the available antifungal agents, routine susceptibility testing has been deemed essential for all laboratories associated with tertiary care medical centers [8, 9].

The increasing incidence of IMIs in the APAC regions has spurred both small- and large-scale surveillance efforts in Australia [12, 16, 17], India [18, 19], Japan [20], Korea [21, 22], Indonesia [23], Thailand [24, 25], Taiwan [14, 26], and several other countries [2, 12, 15]. One of the limitations of the existing surveillance data from the APAC region is that many of these reports are limited to a single institution and most fail to compare results across cities or countries.

The SENTRY Antifungal Surveillance Program is a survey that has been active globally since 1997, reporting on the frequency of pathogen occurrence and pathogen susceptibility to various antifungal agents [27–29]. The SENTRY Program remains one of the only global antifungal surveys that monitors resistance in *Aspergillus* species and other molds. Given the high degree of antifungal resistance in many of the emerging molds, understanding the activity, efficacy, and limitations of the available antifungal agents is critical for the management of these potentially life-threatening infections [6, 9–11].

One of the important features of the SENTRY Antifungal Surveillance Program is the provision of reference quality fungal identification and antifungal susceptibility testing results to participating laboratories that may lack mycological expertise. This service is dearly needed for APAC laboratories, where identification and antifungal susceptibility testing of filamentous fungi is often lacking and the variability in both species identification and antifungal susceptibility is considerable [2, 8, 9, 12].

In the present study, we summarized the results of the APAC component of the SENTRY Program between 2011 and 2019, comparing the activities of four mold-active triazoles tested against a collection of 372 invasive molds, including *Aspergillus* spp (318 isolates), Mucorales (13 isolates), *Scedosporium* spp (17 isolates), and 12 different species of other rare molds (23 isolates). All isolates were tested using the reference broth microdilution (BMD) method as recommended by the Clinical and Laboratory Standards Institute (CLSI). Emerging resistance was evaluated by species-specific epidemiological cutoff values (ECVs), where available.

## Materials and methods

### Organisms

A total of 372 non-duplicate clinical isolates of molds were collected in 17 hospitals located in six Asia-Pacific countries during a 9-year period (2011–2019). Isolates were recovered from patients with bloodstream infections (9 isolates), pneumonia in hospitalized patients (262 isolates), skin and skin structure infections (24 isolates), and from other non-specified sites of infection (77 isolates).

### Identification methods

Mold isolates were submitted to JMI Laboratories (North Liberty, Iowa, USA), where identification was confirmed by morphological, biochemical, MALDI-TOF MS as well as molecular methods when necessary [11, 30]. Mold isolates were subcultured to assess purity and viability, then inoculated into Sabouraud Liquid Broth Modified (Becton, Dickenson and Company, Sparks, Maryland, USA). Total protein extraction was performed using formic acid and then submitted to MALDI-TOF MS using the MALDI Biotyper (Bruker Daltonics, Billerica, Massachusetts, USA). Isolates not scoring ≥2.0 by spectrometry were submitted to 28S ribosomal subunit sequencing, followed by analysis of β-tubulin (*Aspergillus* spp.), translation elongation factor (TEF; *Fusarium* spp.), or internal transcribed spacer regions (all other species of filamentous fungi), [11, 15, 30, 31]. Nucleotide sequences were analyzed using Lasergene^®^ software (DNASTAR, Madison, Wisconsin, USA) and compared to sequences using BLAST (https://blast.ncbi.nlm.nih.gov/Blast.cqi). TEF sequences were analyzed using the *Fusarium* multilocus sequence typing (MLST) database (http://www.westerdijkinstitute.nl/fusarium/).

### Susceptibility testing

All mold isolates were tested by BMD as described by the CLSI M38 document [32]. Frozen-form microdilution panels using RPMI 1640 broth supplemented with MOPS (3-[N-Morpholino]propane sulfonic acid) and 0.2% glucose were inoculated with 0.4–5.0 × 10^4^ CFU ml^−1^ conidial suspensions for a final concentration of 0.2–2.5 × 10^4^ CFU ml^−1^. Minimal inhibitory concentration (MIC) endpoints were read at the lowest concentration that produced visually clear wells after 24 h (*Mucorales* group), 48 h (*Aspergillus* spp., other molds), and 72 h (*Scedosporium* spp.). Isavuconazole was included in the SENTRY Program in 2017.

CLSI clinical breakpoints have only been established for voriconazole against *Aspergillus fumigatus*; however, ECVs have been developed by CLSI for isavuconazole, itraconazole, posaconazole, and voriconazole against *Aspergillus flavus* species complex (SC)*, Aspergillus terreus* SC, and *Aspergillus niger* SC, and for isavuconazole and itraconazole against *A. fumigatus* SC [33–36]. Isolates of *Aspergillus* spp. for which the triazole MIC results exceeded the ECV are considered to be non-wildtype (NWT) and may harbor acquired mutations in the *cyp51A* gene [6, 7, 27, 35–38].

### Quality control

Quality control (QC) was performed in accordance with CLSI guidelines using *A. flavus* ATCC 204304 and *A. fumigatus* ATCC MYA-3626 [32]. All MIC values were within their respective QC ranges [39].

## Results

### Activity of mold-active azoles against *Aspergillus* spp. from APAC, 2011–2019

A total of 318 clinical isolates of *Aspergillus* spp. were tested in the surveillance years of 2011–2019 and are presented in Table 1. Isolates were obtained from six countries, including Australia (164 isolates), Thailand (79 isolates), China (34 isolates), Korea (26 isolates), New Zealand (13 isolates), and Singapore (2 isolates). During the study period, 14 different species or SC were identified, including: *A. fumigatus* (189 isolates), *A. flavus* SC (43 isolates), *A. niger* SC (46 isolates), *A. terreus* SC (14 isolates), *A. nidulans* SC (11 isolates), *A. tamarii* (3 isolates), *A. versicolor* (3 isolates)*, A. tubingensis* (2 isolates), *A. lentulus* (2 isolates), and single isolates of *A. aculeatus, A. clavatus, A. foetidus*, *A. ochraceus* SC, and *A. ustus SC* (Table 1).

**Table 1:** Geographic distribution of *Aspergillus* spp. collected during 2011–2019 in Asia and Western Pacific Region medical centers participating in the SENTRY Antifungal Surveillance Program

Organism/organism group^a^	Australia	China	Korea	New Zealand	Singapore	Thailand	Total
All *Aspergillus* spp.	164	34	26	13	2	79	318
*A. fumigatus*	113	20	6	11	1	38	189
*A. flavus* SC	4	9	9			21	43
*A. niger* SC	24	4	6			12	46
*A. terreus* SC	6	1	2		1	4	14
*A. nidulans* SC	11						11
*A. tamarii*	1			1		1	3
*A. versicolor*	1		2				3
*A. tubingensis*	1		1				2
*A. aculeatus*						1	1
*A. clavatus*						1	1
*A. foetidus*	1						1
*A. lentulus*	1			1			2
*A. ochraceus* SC						1	1
*A. ustus* SC	1						1

^*a*^*SC* species complex

The in vitro activities of the four mold-active triazoles against *Aspergillus* spp. are shown in Table 2. Similar activities were observed when isavuconazole (MIC_50_/_90_, 0.5/2 mg l^−1^), itraconazole (MIC_50_/_90_, 0.5/1 mg l^−1^), and voriconazole (MIC_50_/_90_, 0.5/1 mg l^−1^) were tested against *Aspergillus* spp. isolates. Those activities were 1–2-fold dilutions higher than posaconazole (MIC_50_/_90_, 0.25/0.5 mg l^−1^). *A. fumigatus* displayed MIC_90_ values of 1 mg l^−1^for isavuconazole and itraconazole and 0.5 mg l^−1^ for posaconazole and voriconazole (Table 2). Most of the *A. fumigatus* isolates tested were WT to isavuconazole (94.3% [CLSI ECV]), itraconazole (97.9% [CLSI ECV]), and voriconazole (98.9% [CLSI ECV]). In addition, the voriconazole susceptibility rate against *A. fumigatus* was 95.8% when the CLSI breakpoint was applied. Posaconazole does not have a CLSI-published ECV criteria against *A. fumigatus*, but there has been discussion of whether the ECV should be 0.25 or 0.5 mg l^−1^ [40]. If the ECV for posaconazole were to be set at 0.5 mg l^−1^, 98.9% of *A. fumigatus* isolates in this collection would be WT. The overall frequency of NWT strains of *A. fumigatus* was 1.1% for posaconazole and voriconazole, 2.1% for itraconazole, and 5.7% for isavuconazole. All NWT strains originated from either Australia or Thailand (data not shown).

**Table 2:** Antifungal activity of isavuconazole and comparator antifungal agents against *Aspergillus* spp. collected during 2011–2019 in APAC medical centers participating in the SENTRY Antifungal Surveillance Program

Species	Antifungal agent (no. tested)	MIC (µg ml^−1^)			ECV	
		Range	50%	90%	%WT	%NWT
*Aspergillus* spp.	Isavuconazole (126)	0.015–4	0.5	2		
	Itraconazole (319)	0.12–>8	0.5	1		
	Posaconazole (319)	0.03–>8	0.25	0.5		
	Voriconazole (319)	0.03–4	0.5	1		
*A. fumigatus*	Isavuconazole (70)	0.25–2	0.5	1	94.3	5.7
	Itraconazole (189)	0.25–>8	0.5	1	97.9	2.1
	Posaconazole (189)	0.03–>8	0.25	0.5	98.9^a^	1.1
	Voriconazole (189)	0.06–4	0.5	0.5	98.9	1.1
*A. flavus* SC	Isavuconazole (19)	0.5–2	0.5	1	94.7	5.3
	Itraconazole (43)	0.25–1	0.5	1	100.0	0.0
	Posaconazole (43)	0.12–1	0.25	0.5	97.7	2.3
	Voriconazole (43)	0.12–2	0.5	1	100.0	0.0
*A. niger* SC	Isavuconazole (18)	0.12–4	2	4	100.0	0.0
	Itraconazole (46)	0.12–8	1	2	97.8	2.2
	Posaconazole (46)	0.06–1	0.5	1	100.0	0.0
	Voriconazole (46)	0.06–2	1	1	100.0	0.0
*A. terreus* SC	Isavuconazole (6)	0.06–0.5	0.12		100.0	0.0
	Itraconazole (14)	0.25–1	0.5	1	100.0	0.0
	Posaconazole (14)	0.12–0.5	0.25	0.25	100.0	0.0
	Voriconazole (14)	0.06–0.5	0.25	0.5	100.0	0.0
*A. nidulans* SC	Isavuconazole (6)	0.015–0.25	0.03			
	Itraconazole (11)	0.12–1	0.5	0.5		
	Posaconazole (11)	0.06–0.5	0.25	0.5		
	Voriconazole (11)	0.03–0.5	0.12	0.25		
*A. tamari*	Isavuconazole (2)	0.12–0.25	0.12			
	Itraconazole (3)	0.25–0.5	0.25			
	Posaconazole (3)	0.06–0.25	0.12			
	Voriconazole (3)	0.12–0.5	0.25			
*A. versicolor*	Isavuconazole (1)	0.5				
	Itraconazole (3)	0.5–1	0.5			
	Posaconazole (3)	0.5	0.5			
	Voriconazole (3)	0.25–0.5	0.5			

*SC* species complex

^a^Using an ECV of ≤0.5 mg l^−1^ [40].

The MIC_90_ values were 1 mg l^−1^ for itraconazole, isavuconazole, and voriconazole and 0.5 mg l^−1^ for posaconazole and *A. flavus* SC, with 100.0%, 94.7%, 100.0%, and 97.7% of the isolates considered as WT, respectively (Table 2). The isavuconazole MIC_90_ value of 4 mg l^−1^ for *A. niger* SC (Table 2) was comparable to the MIC_90_ of itraconazole (2 mg l^−1^) and higher than the MIC_90_ of posaconazole (1 mg l^−1^) and voriconazole (1 mg l^−1^). The WT percent for *A. niger* SC was 100.0% for isavuconazole, posaconazole, and voriconazole and 97.8% for itraconazole (Table 2). All isolates of *A. terreus* SC were WT to all four triazoles. *A. nidulans* SC, *A. tamarii*, and *A. versicolor* were all susceptible to these agents, with MIC_50/90_ values of 0.03–0.5 mg l^−1^.

There were nine isolates of rare *Aspergillus* species represented by one or two isolates each. These rare *Aspergillus* species included: *A. tubingensis* (2 isolates), *A. aculeatus* (1 isolate), *A. clavatus* (1 isolate), *A. foetidus* (1 isolate), *A. lentulus* (2 isolates), *A. ochraceus* SC (1 isolate), and *A. ustus* SC (1 isolate) (Table 1). The azole MIC values were generally less than 2 mg l^−1^ for each agent and were comparable to the values seen with *A. fumigatus* (data not shown). Isavuconazole MIC values of 2 mg l^−1^ were seen with *A. tubingensis* and *A. lentulus*.

### Activity of mold-active azoles against non-*Aspergillus* molds from APAC, 2011–2019

A total of 53 isolates of non-*Aspergillus* molds were tested in the surveillance years 2011–2019 and are presented in Table 3. Isolates were obtained from four countries, including Australia (33 isolates), Thailand (10 isolates), Korea (5 isolates), and New Zealand (5 isolates). These organisms included 23 species or SC, but most organisms were represented by 4 or fewer strains. In this survey, the most frequent of these uncommon molds were the *Scedosporium* spp (*S. apiospermum* [1 isolate], *S. apiospermum/S. boydii* [9 isolates], and *S. aurantiacum* [7 isolates]) and *Lomentospora prolificans* (6 isolates), all of which were from either Australia or New Zealand (Table 3). The in vitro activity of isavuconazole, posaconazole, and voriconazole against these molds are shown in Table 4. Although the small number of isolates from each species makes it difficult to obtain conclusions regarding the activity of the triazoles, there are some clear patterns. First, none of the triazoles showed activity against *Fusarium solani* SC or *L. prolificans*. The individual species of *Scedosporium* were more susceptible to voriconazole (MIC range 0.12–8 mg l^−1^; MICs ≤ 1 mg l^−1^ for 15/17 isolates) than either isavuconazole (MIC range 1–>8 mg l^−1^; MICs ≥4 mg l^−1^ for 7/8 isolates tested) or posaconazole (MIC range 0.5–>8 mg l^−1^; MICs ≥ 2 mg l^−1^ for 13/17 isolates tested). Thirteen isolates from the Mucorales group were tested in this survey, including *Lichtheimia corymbifera, L. ramosa*, *Mucor circinelloides*, *Rhizomuco*r *pusilus*, *Rhizopus microsporus* group, *R. oryzae* SC, *Cunninghamella* sp., and *Syncephalastrum* sp. The Mucorales group MIC values for posaconazole ranged from 0.5 to 4 mg l^−1^, with MIC values ≤1 mg l^−1^ for 9/13 isolates. Isavuconazole MIC values of 2 mg l^−1^ were seen for *L. corymbifera*, *R. microsporus* group, and *Syncephalastrum* sp., but were >8 mg l^−1^ for isolates of *R. oryzae* SC. Voriconazole was inactive against the Mucorales group. Among the remaining species of rare molds, elevated MIC values (> 8 mg l^−1^) for both isavuconazole and voriconazole were seen in the *Rasamsonia argillacea* and *Paecilomyces* sp. isolates. One isolate of *Purpureocillium lilacinum* was resistant (MIC ≥8 mg l^−1^) to both posaconazole and voriconazole. The triazoles were all active (MIC ≤ 1 mg l^−1^) against isolates of *Curvularia* sp., *Phialemoniopsis* sp., *Pleurostoma richardsiae*, and *Verruconis gallopava*.

**Table 3:** Geographic distribution of non-*Aspergillus* molds collected during 2011–2019 APAC medical centers participating in the SENTRY Antifungal Surveillance Program

Organism/organism group	Australia	Korea	New Zealand	Thailand	Total
*Fusarium solani SC*				4	4
*Lichtheimia corymbifera*	1				1
*L. ramosa*		1	1		2
*Mucor circinelloides*			1		1
*Rhizomucor pusillus*				1	1
*Rhizopus microsporus*		2			2
*R. oryzae* SC	3			1	4
*Cunninghamella sp*.	1				1
*Syncephalastrum* sp.				1	1
*Scedosporium* spp^a^	15		2		17
*Exophiala dermatitidis*	1				1
*Lomentospora prolificans*	6				6
*Purpureocillium lilacinum*	1			1	2
*Pleurostoma richardsiae*				1	1
*Rasamsonia argillacea*	1				1
*Trichoderma longibrachiatum*	1				1
*Curvularia* spp.	2				2
*Paecilomyces* spp.		2			2
*Phaeoacremonium* sp.	1				1
*Phialemoniopsis* sp.				1	1
*Verruconis gallopava*			1		1
Total	33	5	5	10	53

^a^Includes *S. apiospermum* (1 isolate), *S. apiospermum*/*S. boydii* (9 isolates), and *S. auriantiacum* (7 isolates)

**Table 4:** Antifungal activity of isavuconazole and comparator antifungal agents for non-*Aspergillus* mold isolates collected during 2011–2019 in Asia-Western Pacific medical centers participating in the SENTRY Antifungal Surveillance Program

Organism	MIC results in mg l^−1a^		
	Isavuconazole	Posaconazole	Voriconazole
*Fusarium solani species* complex	>8	>8	>8
*Fusarium solani species* complex	>8	>8	4
*Fusarium solani species* complex	>8	>8	8
*Fusarium solani species* complex	NT	>8	>8
*Lichtheimia corymbifera*	2	0.5	>8
*Lichtheimia ramosa*	NT	1	>8
*Lichtheimia ramosa*	NT	0.5	>8
*Mucor circinelloides*	NT	2	>8
*Rhizomucor pusillus*	NT	0.5	>8
*Rhizopus microsporus* group	2	2	>8
*Rhizopus microsporus* group	2	0.5	>8
*Rhizopus oryzae* SC	>8	1	>8
*Rhizopus oryzae* SC	>8	2	>8
*Rhizopus oryzae* SC	>8	4	>8
*Rhizopus oryzae* SC	NT	0.5	>8
*Cunninghamella* sp.	8	1	>8
*Syncephalastrum* sp.	2	0.5	>8
*Scedosporium apiospermum*	NT	1	1
*Scedosporium apiospermum/S. boydii*	NT	1	0.12
*Scedosporium apiospermum/S. boydii*	NT	0.5	0.5
*Scedosporium apiospermum/S. boydii*	4	1	0.5
*Scedosporium apiospermum/S. boydii*	4	2	0.5
*Scedosporium apiospermum/S. boydii*	4	4	0.5
*Scedosporium apiospermum/S. boydii*	NT	2	1
*Scedosporium apiospermum/S. boydii*	>8	2	1
*Scedosporium apiospermum/S. boydii*	8	2	4
*Scedosporium apiospermum/S. boydii*	1	>8	8
*Scedosporium aurantiacum*	NT	2	0.5
*Scedosporium aurantiacum*	NT	2	0.5
*Scedosporium aurantiacum*	8	2	0.5
*Scedosporium aurantiacum*	8	2	0.5
*Scedosporium aurantiacum*	NT	2	1
*Scedosporium aurantiacum*	NT	2	1
*Scedosporium aurantiacum*	NT	2	1
*Exophiala dermatitidis*	1	0.25	0.25
*Lomentospora prolificans*	NT	>8	8
*Lomentospora prolificans*	NT	>8	>8
*Lomentospora prolificans*	NT	>8	>8
*Lomentospora prolificans*	NT	>8	>8
*Lomentospora prolificans*	>8	>8	>8
*Lomentospora prolificans*	>8	>8	>8
*Purpureocillium lilacinum*	0.5	0.5	0.25
*Purpureocillium lilacinum*	NT	>8	8
*Pleurostoma richardsiae*	0.5	0.5	0.5
*Rasamsonia argillacea*	>8	2	>8
*Trichoderma longibrachiatum*	NT	2	0.5
*Curvularia* sp.	0.5	0.25	0.5
*Curvularia* sp.	1	0.5	0.5
*Paecilomyces* sp.	>8	0.25	>8
*Paecilomyces* sp.	>8	0.25	>8
*Phaeoacremonium* sp.	2	0.5	0.5
*Phialemoniopsis* sp.	0.5	0.25	0.25
*Verruconis gallopava*	1	0.06	0.25

^*a*^*NT* not tested

## Discussion

The majority of IMI are a result of the so-called Big Five mold killers of humans [3]: *Aspergillus, Fusarium, Lomentospora, Scedosporium*, and the Mucorales. Although the epidemiology of IMI is not well described in the APAC region, surveys indicate a rising incidence of infections due to the Big Five [2, 14]. In the present survey, we noted the prominence of *Aspergillus*, *Lomentospora*, *Scedosporium*, and the Mucorales among isolates causing IMI from the APAC region in the SENTRY Antifungal Surveillance Program (Tables 1 and 3). Significantly, an additional 12 different species of rare molds were characterized in this survey, facilitated by MALDI-TOF MS and DNA sequence analysis for accurate organism identification (Table 3).

As expected, *A. fumigatus* was the leading pathogen overall (Table 1). In contrast to the high level of resistance to the triazoles reported from Europe [5, 6, 13, 36], greater than 94% of the APAC isolates were WT to the mold-active triazoles, isavuconazole, itraconazole, posaconazole, and voriconazole (Table 2). We also identified an additional 13 different species of *Aspergillus*, which accounted for 40.6 of the total *Aspergillus* isolates in the collection (Table 1). Although the number of each of these rare species is small, it is important to document their occurrence and antifungal susceptibility profile as the data in the literature are quite limited. As with *A. fumigatus*, the great majority of these non-*fumigatus* species appear to represent WT strains with little acquired resistance to the triazoles (Table 2).

Whereas the *Aspergillus* species in Table 2 appear to maintain susceptibility to the triazoles, this is not the case with most of the non-*Aspergillus* molds (Table 4). Diagnosis of infection with these miscellaneous fungal pathogens seems to be increasing both worldwide and in the APAC region [2, 3, 9, 10, 12, 14, 41]. This finding may be due to the increased identification of clinical isolates of molds using MALDI-TOF MS or DNA sequence analysis, but it also could be attributed to both the immunodeficient state of patients in the region, often complicated by cavitary tuberculosis [1, 21, 24, 26], and the innate resistance of these organisms to antifungal agents [9, 14, 41].

Among the rare molds identified in the present survey, the most common were Mucorales, *Scedosporium*, and *Lomentospora* (Table 3). As observed previously [2, 12, 16], *Scedosporium* and *Lomentospora* were almost exclusively found in Australia (Table 3). Isolates of *L. prolificans* were pan-azole-resistant whereas *Scedosporium* spp. isolates were most susceptible (MIC_50/90_, 0.5/1 mg l^−1^) to voriconazole (Table 4). The Mucorales were represented by eight different species, all of which were resistant (MIC ≥ 8 mg l^−1^) to voriconazole (Table 4). Posaconazole was the most potent triazole against the Mucorales, with MICs ranging from 0.5 to 4 mg l^−1^ (Table 4). Isavuconazole showed activity (MIC 2 mg l^−1^) against *Lichtheimia corymbifera*, *Rhizopus microsporus* group, and *Syncephalastrum* sp., but was not active (MIC ≥8 mg l^−1^) against *Rhizopus oryzae* (Table 4). These findings highlight the need for both accurate identification and antifungal susceptibility testing to optimize the treatment of infections due to the Mucorales [3].

Among the remaining fungi, *Fusarium solani* SC was resistant (MIC ≥ 4 mg l^−1^) to all the tested azoles and *Rasamsonia argillacea* and *Paecilomyces* sp. were resistant to isavuconazole and voriconazole (Table 4).

Isolates of *Curvularia, Phaeoacremonium, Phialemoniopsis*, and *Verruconis gallopava* were generally susceptible (MIC ≤ 1 mg l^−1^) to isavuconazole, posaconazole, and voriconazole (Table 4).

Although many patients with IMI have traditional predisposing factors, such as immunosuppression due to hematological malignancy, blood and marrow transplantation, and solid organ transplantation, these infections are being reported increasingly in non-immunosuppressed individuals [1, 3, 12, 42–44].

Most recently, IMI has been shown to complicate the course of respiratory viral infections, including SARS-CoV-2 and influenza [42–44]. Emerging at-risk populations include those with chronic lung disease (patients receiving steroids and TNF antagonists) and traumatic injuries [3]. Significantly, in the APAC region, both invasive pulmonary aspergillosis and chronic forms of aspergillosis are seen in the setting of cavitary tuberculosis [1, 21, 24, 26]. Traumatic implantation of the non-*Aspergillus* molds, especially the Mucorales and *Scedosporium* spp., is another route of infection in immunocompetent individuals [3, 12].

Given the lack of azole resistance among isolates of *Aspergillus* from the APAC region, the role of mold-active triazoles as first-line agents in the treatment and prophylaxis in high-risk individuals is confirmed [36, 45, 46]. The wide variation in species and antifungal resistance profiles seen among the non-*Aspergillus* molds pose considerable difficulties in patient management [9]. Management of these infections is complicated and prolonged [3]. Extended antifungal therapy, often with two or more agents [9, 47, 48], may be required. Adjunctive surgery is often indicated as a means of source control and to decrease the organism burden [9, 12, 14].

In summary, we have documented the prominence of *Aspergillus* spp. as a cause of IMI in the APAC region, and these isolates remain susceptible/WT to the mold-active triazoles. As the most recently introduced azole, isavuconazole has been shown to be very active against *Aspergillus* spp., including the lesser-known non-*fumigatus* species (Table 2). We have documented the in vitro activity of isavuconazole against *Aspergillus* spp. since 2010 with no change in the MIC distribution over 9 years [10, 11, 15, 28, 31].

In contrast to *Aspergillus* spp., the less common opportunistic molds show a great deal of variety in species and associated resistance profiles [9, 12, 14, 41]. Most prominently, isolates of *Fusarium solani* SC, *L. prolificans*, and the Mucorales express resistance to one or more of the mold-active triazoles, complicating the use of these agents empirically [9]. These findings underscore the importance of building mycological expertise in the APAC region [8]. At present, there is almost no access to advanced diagnostic tests (galactomannan, β-d-glucan, or PCR) in many APAC countries and few laboratories perform DNA sequencing (16.9%) or use MALDI-TOF MS (12.3%) for isolate identification [8]. Antifungal testing for molds is performed in only 27% of laboratories in the APAC region. Increased use of biomarkers (e.g., galactomannan or β-d-glucan) may aid in the diagnosis of invasive pulmonary aspergillosis, but application of proteomic and molecular methods for identification and performance of antifungal susceptibility testing will be necessary to address infections with non-*fumigatus* species of *Aspergillus* as well as the non-*Aspergillus* molds [3, 9]. Accurate identification and the broader application of antifungal susceptibility testing are crucial requirements to find the optimal treatment options for patients with IMI as well as for detection of resistance [49].
