# Correction: Antifungal susceptibilities of opportunistic filamentous fungal pathogens from the Asia and Western Pacific Region: data from the SENTRY Antifungal Surveillance Program (2011–2019)

**DOI:** 10.1038/s41429-021-00460-z

**Published:** 2021-08-18

**Authors:** Michael A. Pfaller, Cecilia G. Carvalhaes, Paul Rhomberg, Shawn A. Messer, Mariana Castanheira

**Affiliations:** 1 JMI Laboratories, North Liberty, IA USA; 2 University of Iowa, Iowa City, IA USA

**Keywords:** Drug development, Medical research

Correction to: *J Antibiot*

10.1038/s41429-021-00431-4

The article “Antifungal susceptibilities of opportunistic filamentous fungal pathogens from the Asia and Western Pacific Region: data from the SENTRY Antifungal Surveillance Program (2011–2019)”, written by Michael A. Pfaller, Cecilia G. Carvalhaes, Paul Rhomberg, Shawn A. Messer & Mariana Castanheira, was originally published Online First without Open Access. After publication in volume 74, issue 8, page 519–527 the author decided to opt for Open Choice and to make the article an Open Access publication. Therefore, the copyright of the article has been changed to © The Author(s) 2021 and the article is forthwith distributed under the terms of the Creative Commons Attribution 4.0 International License, which permits use, sharing, adaptation, distribution, and reproduction in any medium or format, as long as you give appropriate credit to the original author(s) and the source, provide a link to the Creative Commons licence, and indicate if changes were made. The images or other third-party material in this article are included in the article’s Creative Commons licence, unless indicated otherwise in a credit line to the material. If material is not included in the article’s Creative Commons licence and your intended use is not permitted by statutory regulation or exceeds the permitted use, you will need to obtain permission directly from the copyright holder. To view a copy of this licence, visit http://creativecommons.org/licenses/by/4.0.

